# One-year Prevalence, Comorbidities, and Cost of Hospitalizations for Alpha-1 Antitrypsin Deficiency among Patients with Chronic Obstructive Pulmonary Disease in the United States

**DOI:** 10.36469/9799

**Published:** 2017-07-21

**Authors:** Christopher M. Blanchette, Emily Zacherle, Joshua M. Noone, Bryce A. Van Doren, Debosree Roy, Reuben Howden

**Affiliations:** 1 University of North Carolina, Charlotte NC, USA

**Keywords:** hospitalization, copd, chronic obstructive pulmonary disease, aatd, alpha-1 antitrypsin deficiency

## Abstract

**Objectives:** Little is known about severe chronic obstructive pulmonary disease (COPD) exacerbations among patients with Alpha-1 Antitrypsin Deficiency (AATD). We assessed inpatients with AATD and COPD among a sample of COPD inpatients to ascertain demographic, clinical and economic differences in the course of disease and treatment.

**Methods:** Using data from the 2009 Nationwide Inpatient Sample (NIS), we identified COPD (ICD-9-CM: 491.xx, 492.xx, or 496.xx) patients with AATD (273.4). We compared patient demographics and healthcare outcomes (eg, length of stay, inpatient death, type and number of procedures, and cost of care) between COPD patients with and without alpha-1 antitrypsin deficiency. Frequencies and percentages for patient demographics were compared using bivariate statistics (eg, chi-square test). Recognizing the non-parametric nature of length of stay and cost, we calculated median values and interquartile ranges for these variables for each group of patients. Finally, the risk of inpatient death was estimated using logistic regression.

**Results:** Of 840 242 patients with COPD (10.8% of the NIS sample population), 0.08% (684) had a primary or secondary diagnosis code for AATD. COPD+AATD were younger (56 vs 70, p<0.0001) and as a result, less likely to be covered by Medicare (44% vs 62%, p<0.0001). AATD patients were also more likely to have comorbid non-alcoholic liver disease (7% vs 2%, p<0.0001), depression (17% vs 13%, p=0.0328), and pulmonary circulation disorders (7% vs 4%, p=0.0299). Patients with AATD had a 14% longer length of stay (IRR = 1.14, 95% CI 1.07, 1.21) and a mean cost of $1487 (p=0.0251) more than COPD inpatients without AATD.

**Conclusions:** AATD is associated with increased mean length of stay and cost, as well as higher frequency of comorbid non-alcoholic liver disease, depression, and pulmonary circulation disorders. Future research should assess other differences between AATD and the general COPD population such as natural history of disease, treatment responsiveness and disease progression.

## INTRODUCTION

Alpha-I-antitrypsin (AAT), the main protease inhibitor in human serum[1], is produced in the liver and reaches the lungs through circulatory diffusion.[2] In healthy individuals, AAT acts to defend against elastolytic enzymes in the lower airways[3], which are a major contributing factor to emphysema.[4] The release of AAT is up-regulated during inflammation, infection, cancer, and pregnancy.[5] AAT deficiency (AATD) is caused by mutations of the SERPINA I gene encoding AAT-Z[6], and presents with varying ranges of AAT serum concentrations, which has shown to be inversely related to the degree of disease severity. Over 100 alleles and more than 40 phenotypes have been identified, of which null-null and ZZ phenotypes have the lowest serum AAT concentrations, followed by SZ, SS, MZ, MS, and finally MM.[6]

Resulting disease outcomes include pulmonary diseases, such as emphysema and bronchiectasis[7], liver disease (eg, chronic hepatitis, cirrhosis and hepatoma)[8], and, in rare cases, panniculitis.[9] AATD is underrecognized and there are often long delays between symptom onset and diagnosis.[5,10] Two landmark studies conducted in the 1960s described the cardinal features of the disease.[11-13] The first established the genetic predisposition in young adults for early-onset emphysema.[11] The second established the preponderance of early-onset cirrhosis in children with marked decreases in alpha-1 globulin in serum protein electrophoresis patterns.[12]

Caucasians in Europe and North America show the highest risk of mutation and, hence bear the predominant burden of AATD.[14] An estimated 0.11% of Caucasian populations in the United States have AATD.[5,14] An indirect epidemiologic study conducted in 2007 estimated US prevalence of AATD at 33,088 individuals. Combined estimates from population-based screening studies suggest AATD prevalence in US newborns at 1 in 4445 births.[15-17] AATD is usually detected via genetic testing when patients present symptomatic liver and/or lung disease and report a familial history of AATD.[18] This type of exhaustive enquiry is not always performed in all clinical settings and hence AATD continues to be under-recognized and its development is not well understood. Fewer than 10,000 individuals are currently receiving therapy for AAT augmentation, while prevalence estimates suggest this figure should be closer to 70 000 individuals in the United States, which lends support for the assertion that AATD is under-recognized by the medical community.[18]

Chronic obstructive pulmonary disease (COPD) is a progressive, debilitating respiratory disease and includes emphysema and chronic bronchitis.[19] The clinical characteristics of COPD comprise difficulty breathing, lung airflow obstructions, and nonproductive cough.[19] Current prevalence estimates suggest that 6.3% of the US adults have COPD.[19] COPD is influenced by both genetic and environmental factors[20], suggesting AATD may contribute significantly to the pathogenesis of COPD in many patients. Previous studies have reported significant correlations between AATD and pulmonary function.[16,21] Moreover, people with AATD are at an increased risk for developing early-onset COPD.[22]

In this study, we assessed the prevalence of AATD as well as evaluated the demographic, clinical characteristics and cost of hospitalization associated with identifiable and symptomatic AATD in a hospital discharge population with COPD. We also compared COPD patients with AATD to COPD without a recorded AATD diagnosis.

## METHODS

We obtained and analyzed data from the 2009 Nationwide Inpatient Sample (NIS). The NIS contains 100% of the discharge records from 20% of community hospitals in the United States during 2009. The NIS includes information on each discharge, such as patient demographics, hospital characteristics, up to 25 diagnoses, up to 15 procedures, and total charges billed to individual patients. Using the cost-to-charge ratio provided by the NIS, it is possible to estimate the cost of care for each patient encounter. Discharge records in the NIS have been de-identified, so each encounter is treated as a unique event.

We defined our cohort as patients with COPD, limiting our definition of the disease to two conditions: chronic bronchitis and emphysema. The cohort was identified through International Classification of Diseases, Version 9, Clinical Modification (ICD-9-CM) diagnosis codes included in the discharge record. Within this cohort of patients, we identified patients that also had an AATD diagnosis code. Our classification scheme is depicted in Figure 1. (Appendix A includes a list of ICD-9-CM codes used in our classification scheme.)

**Figure 1.** Flow Diagram

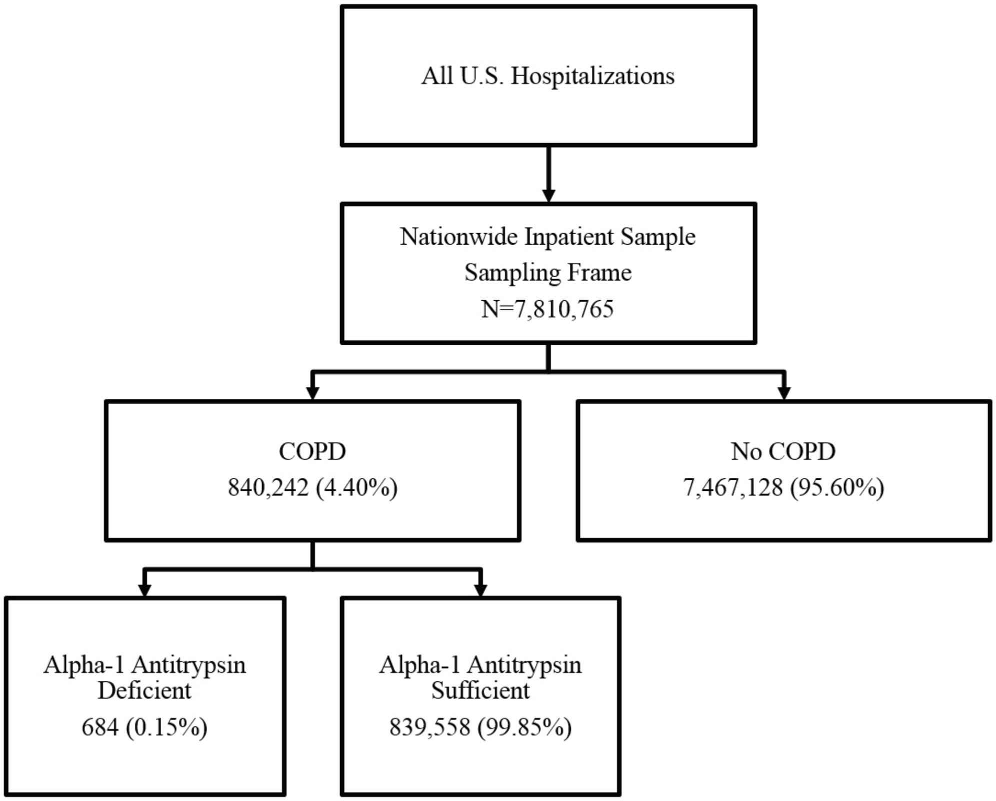

COPD: chronic obstructive pulmonary disease

Since we sought to characterize the population of COPD patients with AATD diagnosis compared to those without a recorded AATD diagnosis, we described the two groups using basic descriptive statistics (eg frequency and proportions) and used bivariate statistics to ascertain differences. We also used multivariable regression models to better assess the incremental effect of AATD on healthcare outcomes while adjusting for patient characteristics, comorbidities, and healthcare use. Using the included sample weights, we calculated national hospitalization incidence estimates for both groups of patients. We compared patient demographics and healthcare outcomes (eg, length of stay, inpatient death, type and number of procedures, and cost of care) between COPD patients with and without AATD. Frequencies and percentages for patient demographics were compared using bivariate statistics (eg, chi-square test for binary and categorical variables, and t-test for continuous variables). As length of stay and cost tend not to be normally distributed, we calculated median values and interquartile ranges for these variables for each group of patients, comparing the values between the groups with the Wilcoxon-Mann-Whitney test. Multivariable models were used to assess the association between AATD and hospital outcomes including length of stay, risk of death and total inpatient cost while adjusting for confounding patient and hospital characteristics. We used a negative binomial regression model to assess length of stay, a logistic regression model to assess risk of inpatient death and a Gamma generalized linear model with log link to assess total inpatient cost. Cost was modeled with the assumption that our continuous outcome data would be non-negative and heteroskedastic.

## RESULTS

Over 4.25 million COPD-related hospitalizations occurred in 2009. We estimated the national hospitalization incidence for COPD with AATD at 3426 patients (Table 1). COPD patients with AATD were roughly 14 years younger, on average, than COPD patients without AATD (55.79 vs 69.77 years, $p<0.0001$). Overall, approximately 4.0% of hospitalizations for COPD, regardless of AAT sufficiency, resulted in death. While fewer than ten COPD patients with AATD were hospitalized with a minor loss of function, 3.6% of COPD patients without a recorded AATD diagnosis entered the hospital with a minor loss of function ($p<0.0001$). COPD patients with AATD were more likely to be white than COPD patients without an AATD diagnosis (76.6% vs 69.7%, $p<0.0001$). Additionally, COPD patients with AATD were more likely to have private health insurance than COPD patients without an AATD diagnosis (23.1% vs 14.3%, $p<0.0001$).

COPD patients with AATD were significantly more likely to have non-alcoholic liver disease than their non-AATD counterparts (7.1% vs 2.2%, $p<0.0001$). However, there was not a significant increase in alcoholic liver disease in AATD patients amongst patients with a reported history of alcohol abuse (17.7% vs 15.9%, $p=0.7436$). Additionally, COPD patients with AATD were less likely to have renal failure (4.0% vs 17.0%, $p<0.0001$) and peripheral vascular disorders (2.0% vs 12.2%, $p<0.0001$). COPD patients with AATD also experienced hypertension (40.0% vs 63.1%, $p<0.0001$) and congestive heart failure (9.8% vs 19.8%) at lower rates than their non-AATD counterparts.

Consistent with the distribution of inpatient deaths reported in Table 1, AATD was not associated with a statistically significant increase in the risk of inpatient death (Table 3) while adjusting for age, sex, region, hospital location (urban/rural), primary payer, loss of function, median household income and race. However, AATD was associated with a 14% increase in length of hospital stay (IRR: 1.14 [1.07-1.21]) (Table 3) and $1487 greater cost ($p=0.0251$) after adjustment.

**Table 1.** COPD Population Characteristics by Alpha-1 Antitrypsin Deficiency Diagnosis

| | COPD with α-1 | COPD without α-1 | χ2 or t-test Significance (p-value) |
|---|---|---|---|
| Hospitalization Incidence Estimate | 3426 | 4 260 171 | - |
| Hospitalizations (n) | 684 | 839 558 | - |
| Inpatient Death (%) | 3.8 | 4.01 | 0.9601 |
| Age (mean) | 55.79 | 69.77 | <0.0001 |
| **Gender (%)** | | | |
| Male | 50.73 | 49.64 | 0.569 |
| Female | 49.27 | 50.36 | |
| **Region (%)** | | | |
| North East | 12.72 | 16.99 | <0.0001 |
| Midwest | 31.58 | 25.17 | |
| South | 37.43 | 41.56 | |
| West | 18.27 | 16.28 | |
| **Hospital Location (%)** | | | |
| Rural | 16.37 | 17.24 | 0.0097 |
| Urban (Non-teaching) | 40.94 | 46.06 | |
| Urban (Teaching) | 40.2 | 34.88 | |
| Not Specified | 2.49 | 1.22 | |
| **Expected Primary Payer** | | | |
| Medicare | 44.15 | 62.43 | <0.0001 |
| Medicaid | 15.64 | 8.74 | |
| Dual Eligible (Medicare & Medicaid) | 12.72 | 9.2 | |
| Private | 23.1 | 14.32 | |
| Other | 4.39 | 5.31 | |
| **Loss of Function (%)** | | | |
| Minor | <1.46 | 3.6 | <0.0001 |
| Moderate | 20.03 | 25.28 | |
| Major | 30.7 | 27.75 | |
| Extreme | 7.46 | 9.55 | |
| Not Specified | 41.52 | 33.82 | |
| **Median Household Income (%)** | | | |
| $1-39 999 | 30.41 | 31.84 | 0.2902 |
| $40 000 - 49 999 | 26.61 | 28.34 | |
| $50 000 - 65 999 | 21.78 | 21.98 | |
| $66 000 or more | 17.84 | 15.39 | |
| Not Specified | 3.36 | 2.46 | |
| **Race (%)** | | | |
| White | 76.61 | 69.71 | <0.0001 |
| Black | 1.9 | 7.93 | |
| Other | 21.49 | 22.36 | |

COPD: chronic obstructive plmonary disease

**Table 2.** Comorbidities by Alpha-1 Diagnosis

| | COPD with α-1 | COPD without α-1 | Significance (p-value) |
|---|---|---|---|
| Hospitalization Incidence Estimate | 3426 | 4 260 171 | - |
| Hospitalizations (n) | 684 | 839 558 | - |
| **Comorbidity (%)** | | | |
| HIV/AIDS | 0 | 0.23 | 0.3377 |
| Alcohol abuse | 4.25 | 5.19 | 0.3988 |
| Alcoholic liver disease* | 17.65 | 15.87 | 0.7436 |
| Deficiency Anemias | 15.5 | 23.1 | 0.0003 |
| Rheumatoid arthritis/Collagen vascular diseases | 3 | 3.17 | 0.8421 |
| Chronic blood loss anemia | <1.46 | 1.46 | 0.1099 |
| Congestive heart failure | 9.75 | 19.84 | <0.0001 |
| Chronic pulmonary disease | 65.5 | 83.93 | <0.0001 |
| Coagulopathy | 5.75 | 4.72 | 0.3322 |
| Depression | 16.75 | 13.14 | 0.0328 |
| Diabetes, uncomplicated | 18 | 26.02 | 0.0003 |
| Diabetes with chronic complications | 2.25 | 5.43 | 0.0051 |
| Drug abuse | 4 | 2.83 | 0.1598 |
| Hypertension | 40 | 63.11 | <0.0001 |
| Hypothyroidism | 10 | 13.38 | 0.0471 |
| Liver disease (non-alcoholic) | 7.05 | 2.16 | <0.0001 |
| Lymphoma | 0 | 0.82 | 0.0858 |
| Fluid and electrolyte disorders | 26 | 28.45 | 0.2769 |
| Metastatic cancer | <1.46 | 2.81 | 0.002 |
| Other neurological disorders | 7.5 | 9.33 | 0.2084 |
| Obesity | 9.75 | 11.8 | 0.2035 |
| Paralysis | <1.46 | 2.39 | 0.0317 |
| Peripheral vascular disorders | 2 | 12.2 | <0.0001 |
| Psychoses | 7.5 | 5.67 | 0.1131 |
| Pulmonary circulation disorders | 6.5 | 4.3 | 0.0299 |
| Pulmonary hypertension | 0 | 0.12 | 0.4384 |
| Renal failure | 4 | 17.02 | <0.0001 |
| Solid tumor without metastasis | 1.75 | 3.62 | 0.0457 |
| Peptic ulcer disease excluding bleeding | 0 | 0.05 | 0.8069 |
| Valvular disease | 1.75 | 5.64 | 0.0007 |
| Weight loss | 6.75 | 6.08 | 0.5756 |

* Limited to patients with history of alcohol abuse. COPD: chronic obstructive pulmonary disease

**Table 3.** Outcomes Associated with AATD

| | Point Estimate | Lower CI | Upper CI |
|---|---|---|---|
| Inpatient Death | OR = 1.36 | 0.89 | 2.07 |
| Length of Stay | IRR = 1.14 | 1.07 | 1.21 |
| Hospitalization Cost | $1487 | p = 0.0251 | |

CI: confidence interval

All estimates are adjusted for age, sex, region, hospital location (urban/rural), primary payer, loss of function, median household income and race.

## DISCUSSION

The current analysis compared demographic and clinical characteristics of a hospital discharge population comprising COPD patients with AATD versus patients with non-AATD associated COPD. Using data from the 2009 Nationwide Inpatient Sample, the results suggest important differences between these patient groups.

COPD inpatients with a diagnosis of AATD have been reported to be younger than COPD inpatients without evidence of AATD.[23,24] In our sample, COPD patients with AATD were approximately 14 years younger than non-AATD CPOD patients. There are two possible explanations for this finding. First, patients with AATD may develop COPD at a much younger age. Previous reports have indicated that the dominant age group of AATD patients with COPD fall into a younger age group than AATD patients without COPD (49.1% aged 59 years or younger compared to 41.9% aged 60 years or older, respectively).[23] Second, patients with AATD may experience more severe symptoms arising from COPD, therefore exacerbations are more likely to occur, prompting the patient to seek medical care. A registry of individuals with severe AATD from March 1989 to October 1992 indicated that AATD participants were more likely to have severe pulmonary function impairment for their age.[25] Furthermore, an examination by Campos et al. found that patients with AATD, despite augmentation therapy (AT), experienced a high number of exacerbations with negative impact on quality of life and health resource utilization (85% of participants).[26] However, without accurate AATD prevalence estimates, and therefore questionable classification of non-AATD associated COPD vs AATD associated COPD, it is difficult to make these claims.

COPD inpatients with a diagnosis of AATD in the current study presented similarly to those of younger, healthier COPD patients when compared to COPD inpatients without AATD. They were more likely to be of working age, to have private insurance and less comorbidities. Conversely, a study by McGrady et al.[27] found that AATD patients reported several comorbidities and with a higher frequency, although more AATD patients reported no comorbidities overall in comparison with COPD patients. However, McGrady's study noted possible recall bias or over/under-reporting, as it was a patient self-reported study.[27]

AATD patients in the current study were more likely to have non-alcoholic liver-disease than their non-AATD counterparts. Research has shown an increase of liver disease in AATD in direct correlation with age and increased risk of cirrhosis and primary liver cancer in patients homozygous for AATD.[29] It is well understood that liver manifestations primarily affect patients with the ZZ genotype (~30% adults, ~2.5% children), although MZ, SZ, and Z-null genotype patients are also at a (low) risk of developing liver disease. Across genotypes, approximately 15% of AATD patients present with liver dysfunction, which may include hepatocarcinoma, cirrhosis, cancer, hepatitis or liver failure.[30,31]

When controlling for age, among other patient and facility characteristics, COPD inpatients with AATD and COPD inpatients without AATD were generally similar in risk of death however experienced a slightly greater length of stay and increased cost. Although no differences were seen in risk of inpatient death, extreme loss of function was highly predictive. Furthermore, AATD patients had increased length of stay in comparison with non-AATD associated COPD patients, with loss of function as a significant predictor. Loss of function is an AHRQ composite measure and is created by using the severity of illness and risk of mortality subclasses of the All Patient Refined Diagnosis Related Groupings Complexity Subclass system (APR-DRG).[32] It is well understood that AATD patients have an increased risk of mortality with certain genotypes, especially PiMZ or PiZZ patients, resulting in more severe lung and/or liver illness.[10] This increase in mortality has been shown to correlate with lung function and CT scan emphysema scores.[33] It has also been reported that AATD patients suffer from more disability even though they are younger and of working age.[27,34-35] Although it is not possible to determine these patient characteristics in the current study, it is possible that the AATD patients studied have more progressed disease, or possibly have the PiMZ or PiZZ type AATD, which therefore accounts for the associations found between extreme loss of function and clinical outcomes in AATD patients.

The current study presents key differences in clinical and economical outcomes between AATD associated COPD and non-AATD associated COPD patients. As AATD is a significantly under-recognized condition, it is essential that further research is done to develop a method for identifying differences in AATD patient characteristics among the standard COPD population. With further insight into these differences, it is possible that we will move closer towards developing a more targeted screening method for identifying high risk patients to avoid delays in diagnosis and under diagnosis of AATD patients worldwide.

## LIMITATIONS

While the NIS is ideal for studying rare diseases it is limited by its observation window and by the nature of discharge data. Any record in the NIS data is for a single hospital stay and cannot be tied to any other record meaning the same individual could be seen more than one time. As each record is a single hospital stay the data is considered discharge data and therefore does not include the actual reimbursed cost by an insurance carrier, but instead has a record of what was charged. These limitations do not impact the overall implications of the results, but do warrant further investigation in other types of datasets.

## CONCLUSIONS

While AATD patients with COPD did not differ from non AATD patients with COPD on inpatient death, they did experience a slightly longer length of stay and increased cost. AATD patients were also quite different based on demographics and comorbidities, suggesting that they may be experiencing similar exacerbations at a much younger age and with different comorbidities.

## DECLARATION OF INTEREST

The authors have no conflicts of interest. This study was not funded, although 4 authors have previously worked as consultants for Grifols USA, LLC, which produces Prolastin-C, a human alpha-1 protease inhibitor indicated for AATD.
